# Factors Influencing Disability and Quality of Life during Treatment: A Cross-Sectional Study on IBD Patients

**DOI:** 10.1155/2019/5354320

**Published:** 2019-08-21

**Authors:** Carla Marinelli, Edoardo Savarino, Marco Inferrera, Greta Lorenzon, Alessandra Rigo, Matteo Ghisa, Sonia Facchin, Renata D'Incà, Fabiana Zingone

**Affiliations:** Department of Surgery, Oncology and Gastroenterology, Gastroenterology Section, University Hospital of Padua, I-35128 Padua, Italy

## Abstract

**Background/Aim:**

Inflammatory bowel disease (IBD) is a chronic disorder affecting patients' quality of life and increasing their disability. The aim of our study was to evaluate clinical and pharmacological factors associated with impaired quality of life and disability in a large cohort of IBD patients during IBD treatment.

**Methods:**

We consecutively and prospectively recruited all IBD patients referred to the IBD Unit of the “Azienda Ospedaliera” of Padua. Demographics and clinical information were collected, and all patients completed the IBD questionnaire (IBDQ) and the IBD-Disability Index (IBD-DI) questionnaire. A multivariate regression model and Spearman's rank correlation coefficient were applied for detecting IBD-related variables relevant to disability and quality of life.

**Results:**

We included 435 IBD patients. Multivariate regression modelling identified active disease, anaemia, presence of extraintestinal manifestations, and Crohn subtype as independent predictors for both disability and poor quality of life. We observed a strong positive correlation between IBD-DI and IBDQ (*r* = 0.84, *p* < 0.001), while there was no association with ongoing therapy or other clinical features disease-related.

**Conclusions:**

Our study showed that disability and quality of life are both associated with active disease, anaemia, presence of extraintestinal manifestations, and Crohn phenotype while ongoing therapy seems not to be associated with disability and QoL during disease management.

## 1. Introduction

Inflammatory bowel disease (IBD) is a group of chronic relapsing disorders that includes principally Crohn's disease (CD) and ulcerative colitis (UC). Patients with IBD usually suffer from severe abdominal pain, diarrhoea, and fever. About 25% of the patients also suffer from abscesses, fistulas, and stenosis [1, 2]. Therefore, IBD patients experience impairment of health-related quality of life, fatigue, depression, and anxiety [3, 4]. Moreover, disease progression may further reduce patients' quality of life and increase their disability [5, 6].

Considering the complexity of IBD management, its chronicity, and the variability of treatment efficacy, current IBD treatment is aimed not only at relieving symptoms and reducing complications but also at improving patient's quality of life [7, 8]. Pharmacological therapy in IBD depends on disease severity and location and includes both conventional therapies (i.e., aminosalicylates, corticosteroids, and immunosuppressive agents) and biologic treatments targeting a specific inflammatory mediator instead of exerting a larger immune suppression. In this regard, antibodies against TNF*α* and integrin antagonists act regulating inflammatory mechanisms in both UC and CD [1]. Available biologic agents differ for immune target, route of administration, and frequency of drug administration [1, 9].

Nowadays, it is believed that evaluation of quality of life is an important measure of patient-reported outcome, although subjective and mainly related to the limitations imposed by the disease [10]. A useful tool to evaluate quality of life in IBD patients until now has been represented by the IBD questionnaire (IBDQ) [11]. Unfortunately, IBDQ has several limitations since it does not consider some aspects such as history of previous surgery, adverse effects of drugs, and presence of extraintestinal manifestations (EIMs). Moreover, it is not appropriate for patients with ostomy [12–15]. These limitations are not present in the IBD-Disability Index (IBD-DI) which has been recently developed to measure disability in IBD patients [6, 16, 17].

Starting from a primary hypothesis that an adequate treatment and disease management should not only induce steroid-free remission, prevent relapses, and surgeries but also avoid IBD-related disability and improve the patients' quality of life, we aimed to evaluate clinical and pharmacological factors associated with impaired quality of life and disability in a large cohort of IBD patients.

## 2. Methods

### 2.1. Patients

This cross-sectional study has been approved by the Ethics Committee of Padua (protocol number 4197/AO/17) in July 2017. We consecutively and prospectively recruited all IBD patients who visited the IBD Unit of the “Azienda Ospedaliera” of Padua from July 2017 to April 2018. Written informed consent was obtained from each patient included in the study. The study protocol conforms to the ethical guidelines of the 1975 Declaration of Helsinki as reflected in a priori approval by the institution's human research committee. Inclusion criteria were age ≥ 18 years and a confirmed diagnosis of UC or CD based on clinical, endoscopic, and histological examinations, according to international criteria [8], from at least six months.

All participants were informed about the nature, duration, and purpose of the study. Demographics and clinical information were taken from outpatient medical records and/or in collaboration with the patient, and all patients were asked to complete the validated IBDQ and the IBD-Disability Index (IBD-DI) questionnaires. Patients were excluded in case of inability to complete questionnaires, significant psychiatric diagnoses (including dementia), stomas, history of alcoholism, or refusal to sign the informed consent form.

### 2.2. Measuring Instruments

#### 2.2.1. Population Characteristics

Sociodemographic information mainly included age, sex, smoking status (classified in two groups: smoker and nonsmoker/past-smoker), and alcohol intake (classified as no alcohol consumption and mild/moderate consumption).

#### 2.2.2. Clinical Assessment (IBD-Related Variables)

Clinical data collected from patients included duration and location phenotypes of intestinal disease (according to Montreal classification), disease activity assessment (using the partial Mayo Score and Harvey-Bradshaw Index), biochemical analysis of faecal calprotectin, age at symptoms' onset and diagnosis, EIMs, history of previous IBD-related surgery, and medical treatments. Current medical treatments were also collected. In particular, we classified the time of biologic therapy in <6 months, more than 6 months and less than 1 year, more than 1 year and less than 5 years, and >5 years of treatment.

#### 2.2.3. IBD Questionnaire (IBDQ) Score

The validated Italian version of the IBDQ [18] was used to measure quality of life. The IBDQ is a 32-item questionnaire looking for bowel symptoms, systemic symptoms, emotional function, and social function. The total score ranges from 32 (poor quality of life) to 224 (good quality of life). IBDQ total score higher than 170 is usually associated with patients in clinical remission [19].

#### 2.2.4. IBD-DI Score

The IBD-DI consists of 19 items organized in 28 parts, exploring disability across 5 domains of the International Classification of Functioning, Disability and Health (ICF): overall health, body functions, activities and participation, body structures, and environmental factors [16]. The total score on the IBD-Disability Index ranges between -80 (maximum degree of disability) and 22 (no disability). A cut-off of 3.5 was previously identified as the differentiation point for IBD versus healthy controls [20].

### 2.3. Statistical Analysis

Categorical and continuous variables were expressed as frequency and mean ± standard deviation (SD), respectively. First, univariate logistic regression models were used to assess whether demographical and IBD-related variables were related to a pathological IBD-DI (score ≤ 3.5) and IBDQ (≤170). Statistically significant variables in the univariate analyses were then included in a multivariate regression model using the backward elimination model to identify the independent variables. We used Spearman's rank correlation coefficient (95% CI) to analyse the correlations between IBD-DI and IBDQ and the following variables: age, disease latency, disease duration, pMayo score, faecal calprotectin, haemoglobin, and IBDQ and IBD-DI, respectively. All tests were 2 tailed with a significance level set at *p* less than 0.05. STATA 11 software was used to analyse the data.

## 3. Results

### 3.1. Study Population Characteristics

We prospectively recruited 463 patients who consecutively visited the IBD Unit of the “Azienda Ospedaliera” of Padua during the study period: 8 patients were excluded because they did not complete the whole questionnaires, 8 patients had stomas, 2 patients had an active psychiatric disorder, and 10 patients declined participation. Thus, 435 IBD patients were included in the study (203 CD, 232 UC). As reported in Table 1, 246 patients were aged more than 45 years. Sex distribution was 204 females and 231 males. In 103 patients, disease was considered clinically active (pMayo > 1 or HBI > 4).

At the time of the study, 173 patients were on therapy with biologic drugs (71 IBD patients with infliximab, 40 with adalimumab, and 49 with vedolizumab; and 13 UC patients with golimumab). The mean time of biologic treatment was 2.1 years ± 2.67 (range 3 months-18 years).

Patients under treatment with immunosuppressants (azathioprine/AZA or methotrexate) were 54, with 27 out of them were on combined therapy (AZA plus biologic therapy, “combo therapy”). Most of the patients who were not on biologic or immunosuppressant therapy took 5-aminosalicylic acid (5-ASA); only a minority (25 subjects) did not receive any treatment. Overall, 109/435 patients had EIMs (Table 1).

### 3.2. Quality of Life Data according to IBDQ

As described in Table 1, from the univariate analysis, we observed that patients with UC had a lower risk of having a pathological IBDQ ≤ 170 (OR 0.59; 95% CI 0.40-0.88) compared to CD patients. IBD patients with active disease were six times more likely to have an IBDQ lower than 170 (OR 5.62; 95% CI 3.47-9.12). Similarly, patients with a high level of calprotectin (>250 *μ*g/g) [21] were approximately two times more likely to have pathological IBDQ scores (OR 1.90; 95% CI 1.28-2.82), and patients with anaemia were approximately three times more likely to have a lower score of IBDQ (OR 2.69; 95% CI 1.68-5.80). Also, patients who consumed alcohol had a lower risk of having an IBDQ score ≤ 170 (OR 0.63; 95% CI 0.42-0.92). Finally, IBD patients with EIMs were four times more likely to have an IBDQ score lower than 170 (OR 3.89; 95% CI 2.47-6.14). Being on biologic therapy was not related to the quality of life (OR 1.42; 95% CI 0.96-2.10). However, multivariate regression using the backward elimination model identified the following factors as independent predictors of poor quality of life: active disease (OR 5.67; 95% CI 3.30-9.76), anaemia (OR 2.15; 95% CI 1.15-4.03), and presence of extraintestinal manifestations (OR 3.15; 95% CI 1.92-5.18). Moreover, this analysis showed that patients with UC had a lower risk of having a pathological IBDQ (OR 0.44; 95% CI 0.28-0.70) (Table 1).

### 3.3. Disability Data according to IBD-DI

Table 2 describes factors influencing the IBD-DI score. From the univariate analysis, we observed that UC patients have a lower risk of having a pathological IBD-DI (OR 0.66; 95% CI 0.45-0.97). Occasional alcohol consumption resulted in a reduced risk of having a pathological IBD-DI score (OR 0.63; 95% CI 0.43-0.93). Furthermore, patients with active disease were five times more likely to have an IBD-DI score lower than 3.5 (OR 5.83; 95% CI 3.41-9.95). Similarly, patients with abnormal calprotectin levels (>250 *μ*g/g) [21] were 1.69 times more likely to have pathological IBD-DI scores (95% CI 1.15-2.48). Patients with anaemia were approximately three times more likely to have a lower score of IBD-DI (OR 2.70; 95% CI 1.50-4.85). Furthermore, IBD patients with EIMs were three times more likely to have an IBD-DI score lower than 3.5 (OR 3.41; 95% CI 2.12-5.48) (Table 2). Being on biologic therapy was not related to the disability (OR 1.31; 95% CI 0.89-1.93).

However, using backward elimination as the multivariate model, we identified the following as independent predictors of disability: active disease (OR 5.62; 95% CI 3.16-9.98), anaemia (OR 1.96; 95% CI 1.02-3.73), and presence of extraintestinal manifestations (OR 2.73; 95% CI 1.65-4.53). Moreover, we observed that patients with UC had a lower risk of having an IBD-DI score ≤ 3.5 (OR 0.52; 95% CI 0.34-0.80) (Table 2).

### 3.4. Biologic Therapy, Disability, and Quality of Life

As shown in Table 3, we focused on patients having a biological therapy ongoing at the moment of the visit (*n* = 173). Univariate analysis showed no-relationship between the time of biologic therapy and the risk of having pathologic IBDQ score or IBD-DI score ≤ 3.5. Moreover, both quality of life and disability were not associated with the combo therapy (AZA plus biologic therapy), with a type of biologic therapy (anti-TNF*α* or anti-integrin) or with the route of administration (intravenous or subcutaneous) in IBD patients.

### 3.5. Multiple Correlation Analysis

In Table 4, we reported results from Spearman correlation analysis.

We observed a negative correlation between IBDQ score and pMayo (*r* = −0.587, *p* < 0.001) and HBI (*r* = −0.63, *p* < 0.001) and between IBDQ and calprotectin (*r* = −0.15, *p* = 0.002). Instead, there was a moderate positive correlation between IBDQ and the haemoglobin (Hb) (*r* = 0.23, *p* < 0.001).

We observed a negative correlation between IBD-DI total score and pMayo (*r* = −0.58, *p* < 0.001) and HBI (*r* = −0.55, *p* < 0.001) and between IBD-DI and calprotectin (*r* = −0.12, *p* = 0.01). Increased disease activity score or calprotectin level generally reflect worsened disability. Instead, there was a positive correlation between the IBD-DI and Hb value (*r* = 0.2, *p* < 0.001).

**Figure 1:**
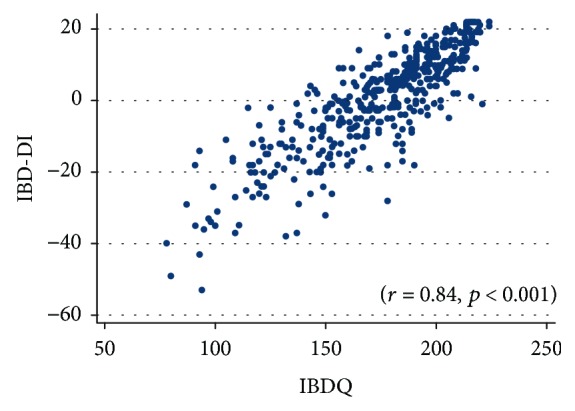
Spearman correlation between IBDQ and IBD-DI scores.

Finally, there was a strong positive correlation between IBD-DI and IBDQ (*r* = 0.84, *p* < 0.001) (Table 4; Figure 1).

## 4. Discussion

Patient-reported outcomes in combination with clinical-reported outcomes have been recently emphasized by medical organizations and regulatory agencies (i.e., FDA and EMA), becoming one of the primary aims of clinical trials focusing to evaluate novel molecules and treatment modalities for IBD patients experiencing disease relapse. In parallel, specific instruments, such as the IBD-DI, have been developed and validated in recent years [16, 17, 20, 22]. Based on these premises, our study was designed to evaluate clinical and pharmacological factors associated with impaired quality of life and disability in a large cohort of IBD patients consecutively enrolled at our IBD outpatient unit.

We prospectively administered the IBD-DI and the IBDQ to a large sample of IBD patients and found that active disease, anaemia, presence of extraintestinal manifestations, and CD were independently related to higher degree of disability, and they were also independently associated with poor quality of life, whereas immunosuppressant and biologic therapy did not play a major role. As expected, we found a strong correlation between disability and poor quality of life.

Clinically active disease was a significant predictor of disability and poor quality of life, as previously demonstrated in literature [17, 20, 22].

Similarly to our results, previous studies focused on IBD-DI questionnaire validation had shown that therapy with biological drugs was not a risk factor for the development of greater disability [17, 22]. Lo et al. found that the disability levels were significantly increased in patients with active disease, treated with systemic steroids, and suffering from EIMs [4]. No relationship was found between current biological therapy and the risk of having pathological score of IBD-DI or poor quality of life, demonstrating that the type of biologic therapy should be chosen based on clinical indications and probability of response only [4].

We found that CD patients scored lower values on IBDQ and IBD-DI than UC patients did, indicating a worse quality of life and increased disability in this IBD subtype. For this reason, CD could be considered as a risk factor of having impaired quality of life and increased disability, in contrast with previous data available in literature that do not find an association with disease phenotype [4, 17, 20, 22]. Moreover, in up to 50% of IBD patients, disease course is associated with EIMs which can worsen during disease progression [23]. Our study demonstrated that EIMs are an independent risk factor for bad quality of life and greater disability in multivariate analysis, in accordance with existing literature [20]. IBD patients often suffer from anaemia, defined as lower levels of haemoglobin [24], because of tissue inflammation. Previous studies have demonstrated that anaemia is strongly correlated with poor quality of life [25], but no data are available on disability correlated with haemoglobin values. We have demonstrated that anaemia is a predictor for bad quality of life and disability. Therefore, disability and quality of life improve as haemoglobin level increases, suggesting that prevention and therapeutic management of anaemia in IBD patients might have a marked impact on reducing patient disability.

We found that disability and quality of life were not related to the type of biologic or the route of administration. In contrast, in a previous study carried out with simple questions, IBD patients demonstrated a preference for intravenous route administration every two months rather than subcutaneous every two weeks [26].

The present study is the first prospective and monocentric study aimed at assessing the factors potentially associated with biological drug administration, disability, and quality of life in a large cohort of IBD patients, followed by the same outpatient unit and according to a standardized protocol. As a second strength point of our study, questionnaires were administered by an interviewer since they were not designed as self-reported questionnaires, in contrast to other similar studies [16, 20]. However, some limitations must be addressed. First, we conducted a cross-sectional study, and thus, we were unable to measure change over time in patient disability and quality of life during the long course of the intestinal disease. Second, the faecal calprotectin level was used as a marker of disease activity since endoscopy was not available for each patient. However, it has been demonstrated to have a good correlation with endoscopic disease severity in both CD and UC patients [27]. Third, despite the large number of patients enrolled, this cohort is not a representative of the whole population; therefore, a larger multinational study should be considered.

In conclusion, our study showed that active disease and its consequence as low values of Hb or the presence of EIM are associated with both disability and quality of life, independently from the ongoing therapy. Therefore, it is mandatory for clinicians to aim for remission in IBD patients. IBDQ and IBD-DI should be regularly assessed together with conventional clinical scores and biochemical tests for a better management of the disease.

## Figures and Tables

**Table 1:** Factors influencing IBD quality of life.

		Univariate analysis	Multivariate model: backward elimination	
Variables	*N* 435	Unadjusted OR *Risk of having pathological IBDQ* (*≤170*)	Adjusted OR *Risk of having pathological IBDQ* (*≤170*)	Adjusted OR *Risk of having pathological IBDQ* (*≤170*)
Disease				
CD	203	1	1	1
UC	232	0.59 (0.40-0.88)	0.44 (0.28-0.71)	0.44 (0.28-0.70)
Age group				
<45	189	1		
≥45	246	1.19 (0.80-1.76)		
Sex				
Men	231	1		
Women	204	1.20 (0.81-1.76)		
Disease latency				
≤1 year	335	1		
>1 year	100	1.03 (0.96-1.11)		
Disease duration				
≤5 years	80	1		
>5 years	355	0.99 (0.97-1.01)		
Current smoking status				
Nonsmoker	371	1		
Smoker	64	0.17 (0.68-2.01)		
Current alcohol intake				
No	189	1	1	
Yes	246	0.63 (0.42-0.92)	0.69 (0.44-1.07)	
Disease active (pMayo > 1 or HBI > 4)				
Inactive	332	1	1	1
Active	103	5.62 (3.47-9.12)	5.47 (3.11-9.61)	5.67 (3.30-9.76)
High faecal calprotectin (>250)				
No	248	1	1	
Yes	187	1.90 (1.28-2.82)	1.18 (0.74-1.87)	
Anaemia (Hb < 12 females, <13 males)				
No	373	1	1	1
Yes	62	2.69 (1.68-5.80)	2.05 (1.09-3.86)	2.15 (1.15-4.03)
UC localization				
E1/E2	123	1		
E3	109	1.34 (0.77-2.33)		
CD behavior				
Nonstricturing, nonpenetrating	104	1		
Stricturing	65	1.01 (0.54-1.89)		
Penetrating	34	1.26 (0.58-2.73)		
Localization				
L1 terminal ileum	48	1		
L2 colon or L3 ileocolon	148	1.19 (0.61-2/29)		
L4 upper or upper+other	7	1.86 (0.37-9.27)		
Extraintestinal manifestations				
No	326	1	1	1
Yes	109	3.89 (2.47-6.14)	3.08 (1.87-5.08)	3.15 (1.92-5.18)
Immunosuppressant				
No	381	1		
Yes	54	1.19 (0.67-2.12)		
Biologics				
No	262	1		
Yes	173	1.42 (0.96-2.10)		
Surgery				
No	320	1		
Yes	115	1.36 (0.88-2.10)		

**Table 2:** Factors influencing IBD-Disability Index.

		Univariate analysis	Multivariate model: backward elimination	
Variables	*N*	Unadjusted OR Risk of having pathological IBD-DI (≤3.5)	Adjusted OR Risk of having pathological IBD-DI (≤3.5)	Adjusted OR Risk of having pathological IBD-DI (≤3.5)
Disease				
CD	203	1	1	1
UC	232	0.66 (0.45-0.97)	0.52 (0.34-0.80)	0.52 (0.34-0.80)
Age group				
<45	189	1		
≥45	246	1.25 (0.85-1.83)		
Sex				
Men	231	1		
Women	204	1.26 (0.86-1.84)		
Disease latency				
≤1 year	335	1		
>1 year	100	1.03 (0.95-1.11)		
Disease duration				
≤5 years	80	1		
>5 years	355	1.00 (0.98-1.02)		
Current smoking status				
Nonsmoker	371	1		
Smoker	64	0.89 (0.52-1.52)		
Current alcohol intake				
No	189	1	1	
Yes	246	0.63 (0.43-0.93)	0.67 (0.44-1.02)	
Disease activity (pMayo or HBI)				
Inactive	332	1	1	1
Active	103	5.83 (3.41-9.95)	5.61 (3.10-10.17)	5.62 (3.16-9.98)
High faecal calprotectin (>250)				
No	248	1	1	
Yes	187	1.69 (1.15-2.48)	1.07 (0.69-1.67)	
Anaemia (Hb < 12 females, <13 males)				
No	373	1	1	1
Yes	62	2.70 (1.50-4.85)	1.88 (0.98-3.62)	1.96 (1.02-3.73)
UC localization				
E1/E2	123	1		
E3	109	1.29 (0.77-2.17)		
CD behavior				
Nonstricturing, nonpenetrating	104	1		
Stricturing	65	1.20 (0.64-2.25)		
Penetrating	34	1.22 (0.55-2.68)		
Localization				
L1 terminal ileum	48	1		
L2 colon or L3 ileocolon	148	1.42 (0.74-2.74)		
L4 upper or upper+other	7	0.74 (0.15-3.71)		
Extraintestinal manifestations				
No	326	1	1	1
Yes	109	3.41 (2.12-5.48)	2.66 (1.59-4.43)	2.73 (1.65-4.53)
Immunosuppressant				
No	381	1		
Yes	54	1.35 (0.76-2.40)		
Biologics				
No	262	1		
Yes	173	1.31 (0.89-1.93)		
Surgery				
No	320	1		
Yes	115	1.50 (0.97-2.31)		

**Table 3:** Focus on biologic and quality of life.

		All	All
Variables	*N* 173	Unadjusted OR *Risk of having pathological IBDQ* (*≤170*)	Unadjusted OR *Risk of having pathological IBD-DI* (*≤3.5*)
Time of therapy			
<6 months	53	1	1
6 months-1 year	20	0.91 (0.32-2.57)	0.89 (0.31-2.49)
1 year-5years	80	0.78 (0.38-1.59)	1.02 (0.50-2.08)
>5 years	20	0.65 (0.22-1.91)	1.53 (0.52-4.49)
Combo therapy^∗^			
No	149	1	1
Yes	27	0.85 (0.37-1.97)	1.23 (0.53-2.83)
Type of biologic			
Infliximab	71	1	1
Adalimumab	40	0.96 (0.43-2.12)	0.74 (0.34-1.61)
Vedolizumab	49	1.28 (0.61-2.66)	1.00 (0.48-2.09)
Golimumab	13	2.31 (0.68-7.79)	2.73 (0.69-10.78)
Type of administration			
Intravenous	120	1	1
Subcutaneous	53	1.08 (0.56-2.07)	1.98 (0.51-1.89)
Type of drug			
Anti-TNF	124	1	1
Anti-integrin	49	1.18 (0.60-2.30)	1.01 (0.51-1.96)

^∗^Combo therapy: AZA plus biologic therapy.

**Table 4:** Spearman correlation analysis between IBD-related variables and IBD-DI and IBDQ scores.

	IBDQ	*p*	IBD-DI	*p*
Age	-0.067	0.16	-0.02	0.58
Disease latency	-0.311	0.52	-0.05	0.22
Disease duration	0.002	0.95	-0.02	0.63
pMayo	-0.587	<0.001	-0.58	<0.001
HBI	-0.63	<0.001	-0.55	<0.001
Calprotectin	-0.15	0.002	-0.12	0.01
Ferritin	0.10	0.057	0.10	0.054
HB	0.23	<0.001	0.2	<0.001
IBDQ	—		0.84	<0.001
IBD-DI	0.84	<0.001	—	

## Data Availability

The data used to support the findings of this study are available from the corresponding author upon request.
